# The Utilization of Algae and Seaweed Biomass for Bioremediation of Heavy Metal-Contaminated Wastewater

**DOI:** 10.3390/molecules27041275

**Published:** 2022-02-14

**Authors:** Hussein Znad, Md. Rabiul Awual, Sri Martini

**Affiliations:** 1 WA School of Mines: Minerals, Energy and Chemical Engineering, Curtin University, GPO Box U1987, Perth, WA 6845, Australia; awual75@yahoo.com; 2 School of Engineering, Edith Cowan University (ECU), Perth, WA 6027, Australia; 3 Chemical Engineering Department, Universitas Muhammadiyah Palembang, Palembang 30263, Indonesia

**Keywords:** heavy metal, algae, seaweed, wastewater, adsorption, adsorbent

## Abstract

The presence of heavy metals in water bodies is linked to the increasing number of industries and populations. This has serious consequences for the quality of human health and the environment. In accordance with this issue, water and wastewater treatment technologies including ion exchange, chemical extraction, and hydrolysis should be conducted as a first water purification stage. However, the sequestration of these toxic substances tends to be expensive, especially for large scale treatment methods that require tedious control and have limited efficiency. Therefore, adsorption methods using adsorbents derived from biomass represent a promising alternative due to their great efficiency and abundance. Algal and seaweed biomass has appeared as a sustainable solution for environmentally friendly adsorbent production. This review further discusses recent developments in the use of algal and seaweed biomass as potential sorbent for heavy metal bioremediation. In addition, relevant aspects like metal toxicity, adsorption mechanism, and parameters affecting the completion of adsorption process are also highlighted. Overall, the critical conclusion drawn is that algae and seaweed biomass can be used to sustainably eliminate heavy metals from wastewater.

## 1. Introduction

Despite the improvement of the economy and the fulfilment of human needs, industrial development has also caused worrying concerns about the availability of clean water due to wastewater disposal in the environment [1,2]. Some of the most threatening constituents in industrial wastewater are heavy metals due to their high toxicity and low degradability in Nature, resulting in serious health consequences to humans and other living organisms. Therefore, it is essential to purify metal ion-polluted water before its final release back to the environment [2,3]. Industries can generate wastewaters containing high loads of heavy metals [4]. Therefore, particular effort must be taken in terms of heavy metals-contaminated water and wastewater treatment in the first place.

Numerous methods have been applied for metal ion removal from solution such as ion exchange, coagulation/flocculation, flotation, photocatalysis, solvent extraction, electroremediation, biological sludge, adsorption, and membrane technology [5,6]. While each method offers both advantages and disadvantages [7], to date, adsorption is still a favored option as it involves a relatively lower energy consumption, easy operation, and high removal efficiency [8]. To support environmental sustainability and circular economy systems, the utilization of low cost biomass sources as adsorbent materials has increased their promising potential [4,9,10]. Among other biomaterials, algae and seaweed have been identified as having competitive efficiency for heavy metal elimination from both simulated and raw wastewater [11]. The use of biomass for metals removal offers various benefits such as high metal uptake, good reusability, cost-effectiveness, wide availability in Nature, and the possibility of their usage in both continuous and batch modes [12,13]. Algae and seaweed biomass-based adsorbents undergo metal ion transport mediated by energy dispersed through the natural cell membranes enabling their efficient removal [14].

Although some articles have already discussed heavy metal removal by adsorbents derived from low-cost materials, the literature shows that reviews of the current trends in the development of adsorbents made of algae and seaweed for water pollution control regarding metal removal are still rare. Therefore, the importance of this study relates to some points such as the comprehensive critical review regarding the utilization of various types of algae and seaweed as heavy metal adsorbents, and a comparative analysis of their performance along with other substantial relevant aspects such as the toxicity and sources of heavy metals, adsorption mechanisma and factors affecting the adsorption process.

## 2. Heavy Metal-Contaminated Water

Water is the most essential substance for all living creatures and acts as an excellent solvent. The existence of harmful pollutants, including heavy metals, in the water will lead to detrimental alteration of aquatic biotic systems. The main sources of heavy metal-contaminated streams are industrial and agricultural sites, followed by domestic sewage. Other than that, certain natural phenomena like land erosion and volcanic eruptions may also add some unwanted metals to open waters [11]. The stubborn bioaccumulation of heavy metals affecting natural food chains has negative impacts on both human health and the broader ecosystem, especially for direct and long-term exposure.

There are various prominent heavy metal pollutants that need to be seriously managed. To begin with, mercury (Hg) is a highly toxic heavy metal with a damaging level of corrosiveness. Its contamination of water bodies also threatens the industrial sector due to its corrosive impact on machinery containing aluminum [15]. In terms of health measures, long-term exposure to this metal can cause neurological disorders and paralysis. Therefore, setting a tolerable limit is a must. In the European Union (EU) the safe values of mercury in drinking water and wastewater are set at 0.001 and 0.005 mg/L, respectively [16,17].

Lead (Pb) metal ion is a heavy metal mostly found in the wastewater streams produced during battery manufacturing and the piping, ceramic and glass industries. Lead oxidation states, namely Pb(IV) and Pb(II), harm human health by triggering damage to the brain, circulatory and nervous systems [18]. Its bioaccumulation in soils can last for for hundreds of years, negatively impacting plant photosynthesis and the food chains in Nature [19]. The World Health Organization (WHO) has set the permissible level of lead in water at 0.05 mg/L [20].

The impact of arsenic (As) metal exposure in its various oxidation states is also harmful [21,22]. This metal can be generated as a by-product of coal combustion and manufacturing processes. As the exposure to arsenic-contaminated water results in fatal diseases like lung dysfunction and cancer [23], the WHO has set the permissible arsenic limit in drinking water at 0.01 mg/L [24].

The contamination of copper (Cu) metal is another issue needing considerable attention as its bioaccumulation is detrimental [25]. While limited trace amounts of copper play a valuable role in medicine and the agricultural industries, its exposure beyond the standardized limit can cause liver damage and lung cancer. The maximum permissible level of copper in water was regulated by the United States Environmental Protection Agency (USEPA) at 1.3 mg/L [26], while other relevant institutions may set different allowable levels.

The existence of carcinogenic cadmium (Cd) metal ion which has high solubility in water merits a serious response [27]. Certain industries like fertilizer production, metal plating, mining, smelting, and fuel combustion form part of its man-made sources. The allowable value of cadmium set by the WHO is around 0.003 mg/L [28].

The next metal pollutant is chromium (Cr). It can be found in the form of Cr(VI) or Cr(III) in industrial waterstreams connected to steel fabrication, the textile and ceramics industries [5,29]. For drinking water, the relevant regulatory permissible limit of this metal is below 0.05 mg/L [8].

Trace amounts of nickel (Ni) metal ion in water are also harmful. It can be detected in various waterstreams related to battery, mining, refining, glass and paint manufacturers [30,31,32]. Cancer-related illnesses can also be triggered by this metal leading to strict regulations on its allowable level in drinking water (0.015 mg/L) [31].

Finally, zinc (Zn) metal ion is also toxic and easily dissolved in water. It is mainly used in the mining, galvanization, and coal industries [33,34]. Even though certain metals like zinc and copper can be considered essential for biochemical reactions in the human body, excess exposure above the stated thresholds could lead to serious health damage. For this reason, relevant international institutions have given guidance regarding the acceptable values of maximum contaminant levels of heavy metals in water and wastewaters, as summarized in Table 1 [4,10,34,35,36].

## 3. Technology for Heavy Metal Removal

### 3.1. Electrochemical Methods

Electrochemistry-related processes for removing metal ions from aqueous media have been considered reliable. The particular mechanism used in this system requires optimizing both anodic and cathodic reactions working in electrochemical cells. The electrical current then is applied as needed for removing metals from water or contaminated solutiona. As a result, the cation will move toward the cathode side, while the anion will be attracted by the anode electrode that could be further flow into a storage tank for subsequent usage. This process is dependable, however, it is relatively costly for large scale industrial application due to the high initial investment cost [37].

### 3.2. Biological Method

Biology-related methods rely on the existence of living microorganisms in the aqueous medium for degrading heavy metals. Even though a longer time is needed to ensure the optimum biological and chemical reactions, and the limited availability of large enough ponds for the process may limit its usage in modern industrial sites, this technique is arguably more environmentally friendly and cost effective [38]. Biological treatment processes can be performed well with the contribution of aerobic, anaerobic, combined aerobic–anaerobic bacteria, fungi or other living microorganisms [39,40,41,42].

### 3.3. Membrane Filtration

Membrane technology mostly applies pressure-driven systems for treating wastewater using the pressure difference between the two sides of an appropriate mebrane. As a physical barrier, membranes separate the feed into two flowing streams based on some specific selection property. Water and wastewater can pass through membrane pores under pressure affected by particle size, initial concentration of the feed, and operating conditions like temperature, pressure and pH. Membranes, which can be categorized into microfiltration, ultrafiltration, nanofiltration, and reverse osmosis ones by considering their pore size, have attracted increasing interest due to their outstanding pollutant removal efficiency [3,43,44]. Other than that, membranes have practical operating procedures, and flexibility in design, however, the initial installation and maintenance cost can be constraints [45]. Another concern when using this technique is its main issue, namely membrane fouling which reduce the performance over time [46].

### 3.4. Coagulation and Flocculation

Coagulation and flocculation work for purifying contaminated water in accordance with the application of flocculant and coagulant agents like aluminum and ferric chloride [47]. In coagulation, the density is increased to destabilize and eliminate the colloidal pollutant particles, leading to further separation of those particles. The next step is the agglomeration of destabilized particles. The flocculation itself may activate heavy metals leading to less removal. Therefore, applying precipitation and spontaneous reduction in the coagulation process can boost metal ion removal. The effectiveness of this process is mostly influenced by the initial pollutant concentration, coagulants and flocculants dosage, and pH [47,48]. Despite the ease of application and good removal efficiency, some side issues including costly chemical reagents and toxic sludge production need further consideration [49].

### 3.5. Advanced Oxidation Processes

Advanced oxidation processes (AOPs) for managing water pollution are able to mineralize organic compounds using hydroxyl radicals [50]. In this category there are some prominent options to perform oxidation processes such as Fenton (H_2_O_2_/Fe^2+^), Fenton-like (H_2_O_2_/Fe^3+^), electron beam irradiation, sonolysis, electrochemical oxidation, photo-assisted Fenton (H_2_O_2_/Fe^2+^/Fe^3+^/UV), and photocatalytic reactions [51,52,53,54]. Like other procedures, these techniques also have some concerning issues like costly chemicals and the production of toxic sludge and other related by-products [55].

### 3.6. Hybrid Methods

Hybrid treatment methods can provide higher removal efficiency overcoming the drawback of each involved method. An example of this idea can be the membrane case study. When considering how to treat high pollutant-loaded wastewaters using only a membrane filtration system, the main issue is quick and severe fouling of the membrane surface. One possible solution to the issue is a pre-treatment process preceding the membrane treatment plant [56,57,58]. Different studies have reported a strategy of combining electrocoagulation, membrane, and ozone techniques for treating oily wastewater, resulting in higher COD removal efficiency. In this work, the elimination of targeted pollutants increased nearly 3-fold compared with a single treatment technique [59].

### 3.7. Adsorption

**Figure 1:**
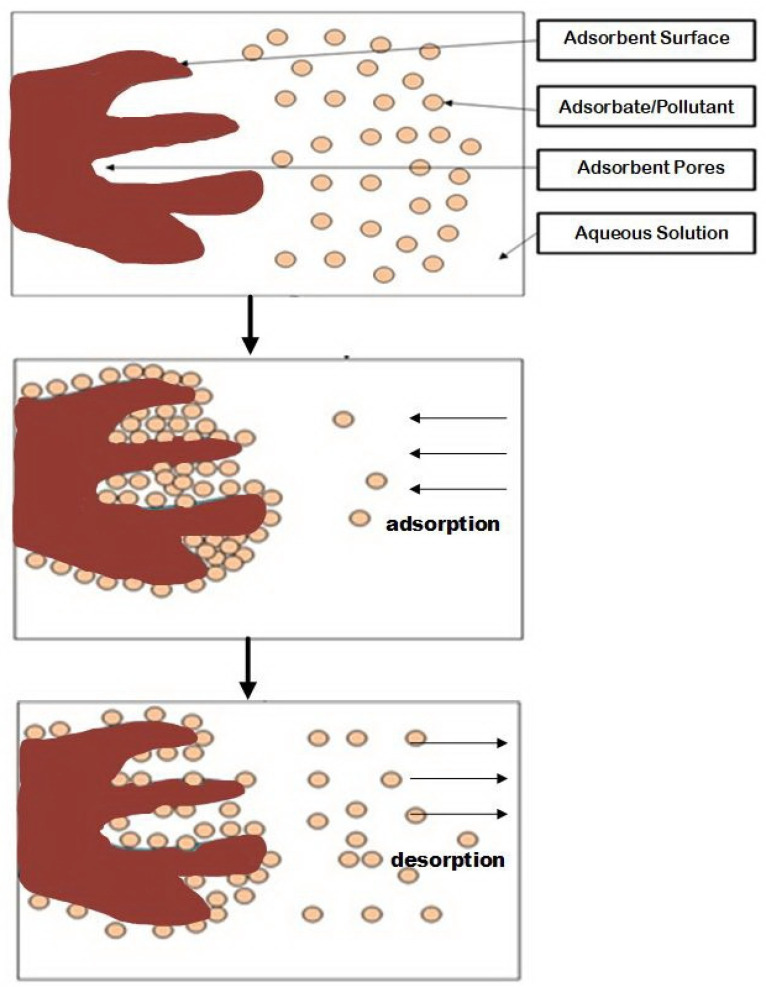
Adsorption and desorption illustration.

Adsorption processes (Figure 1) are among the most prominent methods for treating contaminated solutions. In terms of metals removal, this method involves the accumulation of metal ions onto adsorbent pores and surfaces forming a layer consisting of metal ions, along with other pollutants [6,60]. Adsorption is relatively simple. It has also great removal efficiency, and less energy and chemicals consumption, however, its material preparation and regeneration process should be further concerns [61]. Recently, the utilization of biomass and other natural materials has been increasing due to their potential, eco-friendliness and cost-effectiveness [4]. Various low-cost material-based adsorbents like clays, zeolites, plants and agricultural wastes as well as animal residues have been reported to be competitive and promising [4,62,63,64,65].

## 4. The Influencing Parameters of Heavy Metal Adsorption

The exploration of various natural based-adsorbents has flourished. Bio-adsorbents can experience chemisorption, physisorption, microprecipitation, and oxidation/reduction during the adsorption process [66]. The term chemisorption refers to ion exchange, chelation, and complexation, while physisorption links to electrostatic interactions and van der Waals forces. The adsorbent surface contains functional groups which are crucial for binding metal ions in chemisorption processes. Ion exchange then provides electrostatic interactions between the negatively charged cell walls of the adsorbent and the cations contained in the solution [67]. Furthermore, chelation informs the process of ligand attachment with metal ions forming a ring structure acting as a barrier for the attached minerals to further avoid unwanted chemical reactions [1]. For metal ions, it then connects to the complexation process by forming a complex with the active groups in the cell wall on the cell surface of the adsorbents. The bond formation could be covalent or electrostatic, while the compounds may be neutral, negatively or positively charged [68]. Apart from the aforementioned reactions, the following prominent factors have been suggested as significantly influential points for adsorption processes.

### 4.1. Initial pH

The initial pH has an important influence on the ionization of solute metal ions and the surface chemistry of the adsorbent. In this case, it is essential to perform a zeta potential analysis of the adsorbent to determine the charge of the adsorbent surface. Generally, once the pH solution is lower than the zeta potential, the acidic water will donate more protons than the hydroxide group leading to a positively charged adsorbent surface to better attract the anions. On the contrary, when the surface is negatively charged, it would attract the cation or repel the anion when the pH is higher than the zeta potential value [69,70].

### 4.2. Initial Metal Concentration

The initial concentration of the metal can be a driving force for mass transfer processes between the solute in solution and the adsorbent. During the initial stage, the plot slope tends to be high, and the removal percentage is at a maximum value due to a higher ratio of the initial molar number of metal ions to the active sites of the adsorbent. When the concentration increases, the ratio and the number of available sites will be lower, resulting in decreased removal efficiency [4,10].

### 4.3. Adsorbent Dose

An optimum adsorption capacity is the result of optimum interactions between the active sites of the adsorbent and dissolved metal ions. Most research has showed that the increase in adsorbent dose would provide more sites for trapping or binding metal ions. In other words, the chance of collisions between adsorbent and metal ions is increased leading to better removal efficiency [71]. Some studies utilizing algae-based adsorbents have also reported the significant influence of adsorbent dose where a higher dose could yield higher metal removal to some extent. However, the optimum dose could reach a certain point in which the adsorbent dose above the optimum point would have less or an insignificant effect on the pollutant removal efficiency [10,72].

### 4.4. Contact Time

Contact time is one of the most influential factors affecting the profile of heavy metal removal as well as economic evaluations. The pattern of contact time analysis mostly shows that there is an increasing removal of heavy metals at the beginning of the process. This is caused by the physical adsorption or ion exchange occurring on the surface of a solid adsorbent. At the beginning stage, there are more free binding sites available before they start to decrease and stagnate due to fewer and fewer empty sites for binding metal ions on the surface [10].

### 4.5. Temperature

The temperature level also has to be taken into account for adsorption processes. An increase in temperature would increase metal ions’ tendency to detach from the adsorbent surface. As a result, the functional group bonds would break down, decreasing the forces between metal ions and active sites. In contrast, the higher removal efficiency that occurs at lower temperatures could be attributed to physical adsorption indicating an exothermic mechanism [73,74].

## 5. Adsorption Preparation

**Figure 2:**
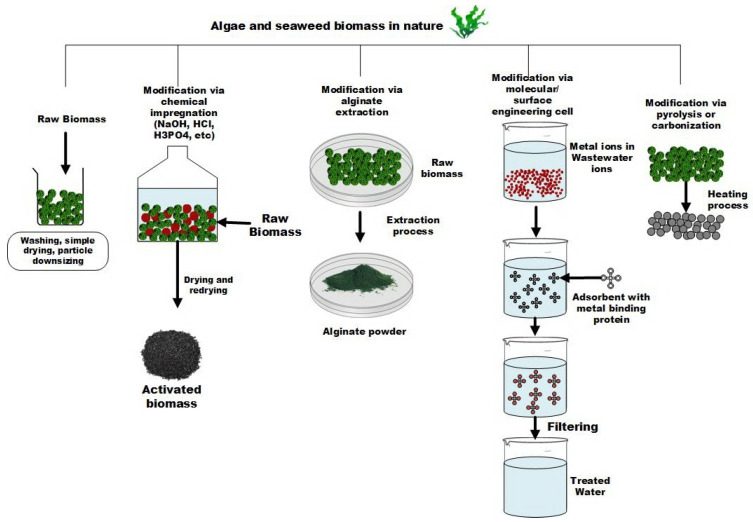
Algae and seaweed-based adsorbent preparation.

Raw biomass should be prepared properly before applying it as an adsorbent. The preparation processes can include physical, thermal, chemical, or combined activation methods. In terms of physical treatment, there are some standard techniques that can be applied such as washing, moderate drying, grinding, or downsizing biomass particles. The range of drying temperature should normally be set below 100 °C considering the natural characteristics of biomass, and this method may not need special techniques like chemical impregnation and magnetization [75]. Therefore, most studies start by synthesizing the bio-adsorbent through some defined physical procedure(s). The next step is thermal or heat treatment. In this case, a high amount of energy is usually needed for activating biomass. Biomass after heat treatment would have a higher surface area and more active sites available on its surface leading to higher adsorption capacity. Generally, pyrolysis processes for biomaterials are conducted at temperatures below 800 °C. This then can be followed by chemical modification to provide more and stronger active sites on the carbonized surface [76]. Chemical treatment that can be implemented as either sole or hybrid activation methods to amplify the affinity of adsorbent to adsorb metal ions through electrostatic interaction correlating to the distribution number of functional groups as well as the surface charge alteration for adsorption improvement [77]. The preparation methods applied for algae and seaweed-based adsorbent are illustrated in Figure 2.

### 5.1. Adsorption Isotherm and Kinetic Models

Adsorption process involves the interaction between an adsorbent (a solid phase) and an aqueous medium containing pollutants. This process may occur in a continuous pattern until it reaches the equilibrium state referring to the amount of adsorbed pollutants and the portion remaining in solution at an equilibrium concentration [10,78]. The mechanistic models may work based on the adsorption mechanism.

#### 5.1.1. Adsorption Isotherm

There are several prominent isotherm models which can be implemented using the experimental data obtained during adsorption processes. They include the Freudlich, Langmuir, Dubinin-Radushkevich, Tempkin, and Elovich isotherm models

- Freundlich Isotherm Model

In this model, the site of adsorption is assumed to occur on the heterogeneous surface of the adsorbent with unequal sites and different adsorption energy levels. The formulation of the Freundlich model is expressed by Equation (1) [79,80]:

(1) $$\ln\;q_{e}=\ln\;k_{f}+(1)/(n)\;(\ln\;C_{e})$$

where $q_{e}$, C_e_, k_f_, and n are the amount of pollutants adsorbed per adsorbent unit at equilibrium (mg/g), the equilibrium of pollutant concentration in solution (mg/L), isotherm constant, and the adsorption intensity, respectively [10].

- Langmuir Isotherm Model

This model refers to an ideal homogeneous and uniform surface on an adsorbent for an adsorption process. In other words, all of the sites on the adsorbent surface are assumed equivalent, which can be expressed using Equation (2) [79]:

(2) $$C_{e}/q_{e}=(1/(K_{L}\;q_{m}))+C_{e}/q_{m}$$

where $q_{m}$ and K_L_ are the maximum adsorption capacity (mg/g) and the Langmuir constant, respectively.

- Dubinin-Radushkevich Isotherm Model

This model points to the Gaussian energy distribution on a heterogeneous adsorbent surface, as expressed in the following linearized formula of Equation (3) [81].

(3) $$\ln\;q_{e}=\ln\;q_{m}-\beta {Ɛ}^{2}$$

where β and Ɛ are a constant related to the adsorption energy and the Polanyi potential regarding the equilibrium concentration, respectively. The Polanyi potential can be obtained from the following equation:

Ɛ = RT ln (1 + 1/C_e_) (4)

where R is the gas constant (8.314 J/mol K), and T is the absolute temperature (K).

- Tempkin Isotherm Model

The Tempkin model believes in the influence of indirect interactions amongst pollutant particles contained in solution during the adsorption process. In addition, the adsorption heat of all molecules available in the layer would decrease linearly with the increase in surface coverage [79]. The linearized formula of the Tempkin model can be written as follows [82]:

(5) $$q_{e}=(\mathrm{RTb})\;\ln\;K_{T}+(\mathrm{RT}/b)\;\ln\;C_{e})$$

where b and K_T_ are the Tempkin constant linking to the heat of adsorption (J/mol) and the Tempkin isotherm constant (L/g), respectively.

- Elovich Isotherm Model

This model holds the principle of kinetics regarding the number of adsorption sites that escalates exponentially along with the adsorption rate indicating a multilayer adsorption pattern, and it can be stated using the following formula (Equation (6)) [82]:

(6) $$\ln\;q_{e}/C_{e}=\ln\;K_{e}\;q_{m}-(q_{e}/q_{m})$$

#### 5.1.2. Adsorption Kinetic Modelling

Kinetic studies are important to understand the relationship between the adsorption mechanism and the contact time. This understanding can be diveded into several related models including the pseudo-first order, pseudo-second order, intra-particle diffusion, liquid film diffusion, and double exponential models. As a result, the probability of adsorbed pollutant particles and external mass transfer, intra-particle mass transfer, and solute diffusivity can be well understood.

- Pseudo-first and Second Order

The pseudo-first-order model links to the adsorption sites on the adsorbent surface occupied by pollutant particles in a number proportional to the number of unoccupied sites. The following equation may allow one to reliably measure it (Equation (7)) [79,80,83,84,85]:

(7) $$\log\;(q_{e}-q_{t})=\log\;(q_{e})-(K_{1}/2.303)\;t$$

where $q_{t}$, K_1_, and t are pollutants adsorbed at a specific time (mg/g), the equilibrium rate constant of pseudo first order adsorption (min^−1^), and time (min), respectively.

The pseudo-second order model meanwhile can be written as follows [85,86]:

(8) $$t/q_{t}{}_{}=1/(K_{2}\;q_{e}^{2})+(1/q_{e})\;t$$

where K_2_ is the pseudo-second order rate constant (g/mg min) estimated by plotting t/$q_{t}$ vs. t. Thus the constant K_2_ can be used to obtain the initial sorption rate (h) at t = 0 (Equation (9)):

(9) $$h=k_{2}\;q_{e}^{2}$$

The values of k_2_, h, and $q_{e}$ can be calculated by the plot of t/q vs. t.

- Intra-particle Diffusion

In this adsorption process, pollutant uptake may vary proportionally with t½ rather than with contact time, as represented by the following equation [79]:

(10) $$q_{t}=K_{id}\;t^{\left(0.5\right)}$$

where *K_id_* and t^(0.5)^ are is the rate constant of intra-particle diffusion (mg/g·min^0.5^) and the square root of time (min), respectively. Plotting $q_{t}$ vs. t^0.5^ then indicates a linear relationship, while the value of *K_id_* can be obtained from its slope.

- Liquid Film Diffusion

The principle of liquid film diffusion relies on the relationship of a liquid and a solid adsorption system in which the rate of accumulated solute in the solid phase of an adsorbent may be equal to that of solute transfer. The following formula then can express this relation Equation (11):

(11) $$\ln\;(1-q_{t}/q_{e})=-K_{fd}\;t$$

where ln (1−$q_{t}$/$q_{e}$) and *K_fd_* are the fractional attainment of equilibrium and the rate constant of film diffusion, respectively. The plot of ln (1 − $q_{t}$/$q_{e}$) vs. t confirms a linear relationship. Additionally, the value of *K_fd_* comes from the slope of the plot.

## 6. Algae or Seaweed-Based Bio-Adsorbents for Sequestering Heavy Metals

Both raw and modified adsorbents derived from algae and seaweed biomass have been reported. Some of the research is summarized in Table 2. In recent years, the efforts to produce novel algae- and seaweed-based adsorbents using extraction, nanoparticles, molecular and chemical modes have achieved remarkable improvements regarding the metal binding capacity and the elucidation of inherent adsorption mechanisms (Figure 2) [87]. Furthermore, heavy metal removal using biomaterials is also influenced by the porosity level and presence of functional groups such as amine, carbonyl, hydroxyl, thiol, carboxyl, and other natural compounds that supporting metal binding like lignins, cellulose, lipids, hydrocarbons, proteins, and tannins. The adsorption capacity and adsorption speed are also affected closely by the exchange capacity of surface cation along with the aforementioned particular operating conditions.

### 6.1. Lead Metal Ions

To begin with, a study has analyzed the feasibility of using algae biomass from the species *Turbinaria ornata* for adsorbing lead from metal-contaminated solutions [88]. After massive washing, the marine algae was dried at 80 °C for 6 h, and then powdered for actual usage. In this work, acidic conditions were found to be effective to remove the targeted metal and the initial metal concentration, adsorbent dose, and the speed of agitation were identified as important factors for success. Under optimum operating conditions, around 99.80% of the lead present could be removed. This happened at an initial concentration of lead of 99.80 mg/L, an agitation speed of 250 rpm and an adsorbent dose of 16.20 g/L. A decreasing lead adsorption trend at higher doses has been noted, confirming the theoretical analysis. In this case, an adsorbent dose beyond the threshold would result in decreasing accumulation of pollutants trapped on the adsorbent active sites.

As the species of the biomass may have various particular properties, another study then investigated another algae species, namely *Gelidium amansii*, as adsorbent by implementing the two-level Plackett–Burman factorial design [89]. After simple preparation steps like washing, drying at 70 °C for around three days, and a downsizing process until the biomass particles had the average size of 125 µm, the prepared adsorbent was applied for adsorbing lead ions from solution. It was then confirmed that the operating parameters like solution pH, metal concentration, and temperature mainly controlled the performance of the adsorbent during the adsorption process. An acidic medium (pH 4.5) was also found optimum for adsorbing lead metal where complete removal could be achieved at an initial lead and adsorbent concentration, temperature, and contact time by 200 mg/L, 1 g/L, 45 °C, and 60 min, respectively. The study utilized functional groups analysis which confirmed the existence of phosphate, carbonyl, phenolic, and methylene groups as important contributors to the excellent adsorption outcome.

### 6.2. Chromium Metal Ions

Chromium is among the heavy metals of concern and hence the subject of serious investigations. Some studies have reported their findings using biomass-based adsorbents. The brown algae known as *Cystoseira barbata* and *Cystoseira crinite*, for example, was concluded to be dependable chromium adsorbents [90]. In the study, both Cr(III) and Cr (VI) could be significantly eliminated from wastewater solutions. Parameters like pH and contact time were identified as the main indicators for adsorption analysis. Firstly, the algal biomass was cleaned several times using distilled water before double drying using natural sunlight and a laboratory oven for around 24 h at 60 °C. The homogenization of biomass particle size was conducted within the range of 100 and 800 μm. Proper contact with wastewater solution containing chromium metal ion was carried out after chemical modification using CaCl_2_ and acetone. The aim of this chemical procedure was to eliminate any proteins and lipids in the hope of increasing the active sites on the adsorbent surface. This study reported that no retention occurred for Cr(VI) ions at an acidic pH of 4.5. Interestingly, while no retention happened for Cr(III) metal ions at an extremely acidic pH of 2.0, that pH value provided the maximum adsorption of sCr(VI) ion. To conclude, Cr(VI) ion could be eliminated from an aquatic environment using these typical algae species. The proton form obtained by harnessing the reducing features of Cr(VI) via electron transfer occurred better in acidic condition for electron donor groups. Around 45% of the Cr(VI) with an initial metal concentration of 100 ppm then could be retained.

Husein then led a study group to continue the exploration of another algae type, namely *Sargassum dentifolium*, for eliminating Cr(VI) metal ions from synthetic wastewater solution [91]. Common preparation treatments like washing, drying in an oven at 50 °C, and size reduction were also applied followed by characterization on specific surface area of adsorbent using Brunauer-Emmett Teller (BET) method according to nitrogen adsorption and desorption isotherms. By applying a factor at a time experiments, the efficiency of Cr(VI) removal could be determined towards the low and high level of each factor. The optimum conditions were recapitulated using a full factorial experimental design. Ultimately, this work claimed the potential of the adsorbent used by informing that around 99.68% of the targeted metal could be adsorbed at an adsorbent dose 1.50 g, particle size 0.3868 μm, and initial metal concentration of 100 ppm. In addition, its reusability was also found promising, as almost 65% of the adsorbed metal could be removed in the first cycle, while the relative stability of regeneration was determined to be good within the first three cycles of the adsorption and desorption process.

### 6.3. Nickel Metal Ions

As one of prominent metal ions detected in various industrial wastewaters, it is worth investigating the potential of biomass-based adsorbents for removing nickel metal from water bodies. A study responded to this idea by utilizing the seaweed species named *Sargassum filipendula* as a nickel adsorbent [92]. Using a dynamic fixed bed system for the adsorption process, an evaluation of flow rates and feed concentrations was performed. Nickel-contaminated solution with an initial concentration of 1.0 mmol/L was flowed at various rates (0.50, 0.80, and 1.0 mL/min) at room temperature to the bottom of the designated column. Initial treatment on the biomass was started by repetitive washing. It was then dried at 60 °C for a day and ground to achieve a particle diameter of around 0.737 mm. Prior to contact with nickel contaminated solution, an alginate extraction procedure was conducted on the powdered biomass with the help of two chemical solutions, namely formaldehyde and hydrochloric acid, followed by rewashing using deionized water. Based on the experiments, around 45 % of the nickel content could be removed at a flow rate and initial metal concentration of 0.5 mL/min and 1.0 mmol/L, respectively. Additionally, regeneration measurements confirmed that even the residue of this activated adsorbent could be reused to remove nickel from solution to some extent even though the regeneration assessment led to the conclusion that the column did not perform further adsorption stages after the second desorption step.

Another work then tried to develop an algal biofilm reactor for removing nickel ions from industrial effluents that have high nickel loadings of up to 5000 mg/L [93]. The microalgae culture was taken care of along with the half of the culture medium being periodically swapped for new Bold’s Basal Medium. The medium consisted of several chemical compounds such as KNO_3_, Na_2_HPO_4_·12H_2_O, MgSO_4_·7H_2_O and FeSO_4_·7H_2_O. The batch mode reactor had a liquid reservoir which was replaced with Ni-contaminated wastewater. The experimental data analysis showed that this algae biofilm reactor system could decrease nickel concentrations in solution by more than 90% yielding a nickel removal rate of 534 mg/L·day. Therefore, it can be concluded that indigenous microalgae grown using this mechanism could purify industrial wastewaters having high loadings of nickel ions.

### 6.4. Cobalt Metal Ions

Water and wastewater containing toxic cobalt ions has to be purified before releasing it to the environment. Bioadsorption can be a great reliable options for this purpose. To overcome the challenges, the potential of a red algae with the species name *Gracilaria corticata* was investigated by Raju et al. [94]. In their work, several relevant parameters were selected and varied, including contact time, initial pH, cobalt ion concentration, adsorbent particle size and dose as well as temperature. Before usage, this red algae was repetitively cleaned and naturally dried under solar light. In order to create a higher adsorbent surface, the particle size of the dried material was further reduced to powder form with various sizes (53, 75, 105, 125 and 152 µm). The equilibrium agitation time needed for adsorbing cobalt ions was 60 min, while the optimum pH was determined to be pH 5, at which up to 87.8% of nickel removal efficiency was achieved. In addition, when the adsorbent dose and temperature by 30 g/L and 303 K, respectively, reported as optimum condition, modelling measurements suggested that the maximum cobalt removal efficiency could be 96.57%, which would happen at pH 4.87, adsorbent dose 31.79 g/L, and initial cobalt concentration 19.77 mg/L. This result was improved when temperature was also increased as indicated by thermodynamic analysis.

A study led by Vafajoo confirmed the significant removal of cobalt (II) metal ions from solution by either raw or modified *Hypnea valentiae*, a species of algae [95]. While raw adsorbent was used directly after standard washing and particle size reduction, the modified version was further treated chemically using a formaldehyde solution. This batch scale investigation mainly focused on the effect of adsorbent dose, metal concentration in solution, and contact time on the adsorbent performance. A maximum adsorption capacity of 10.98 mg/g and 16.66 mg/g for raw and modified algae, respectively, can be achieved under the optimum conditions (temperature 30 °C, initial cobalt concentration of 0.7 mg/L, pH 6 and sorbent dose 2 g/L). In accordance with isotherm analysis, the Langmuir mechanism was believed to be the dominant model during adsorption, while its kinetics followed a pseudo second order model. Apart from that, a study on biomass regeneration indicated a promising reusability rate as the adsorbent could still perform relatively stable even after three adsorption cycles with a maximum capacity of adsorbent regeneration of 66%. For this purpose, the saturated adsorbent was cleaned using 0.1 M of HCl acting as desorbing chemical agent.

### 6.5. Cadmium Metal Ions

For cadmium metal, the potency of algae biomass as an alternative adsorbent has also been documented. To begin with, the algal species *Ulva fasciata* and *Ulva lactuca* were proven promising to reduce the concentration of Cd(II) in solution, even under various culture atmospheres [103]. Despite their simple preparation steps like washing, drying, and grinding, the adsorbents could reach maximum adsorption capacities of 8.353 and 8.804 mg/g for *Ulva fasciata* and *Ulva lactuca*, respectively. Characterization analysis via Fourier transform infrared spectroscopy showed the presence of prominent functional groups such as alkali hydroxyl, amide hydrogen bonda, and carbonyl groups. benefitting the binding force of the adsorbent active sites.

Other work then conducted an evaluation on the extraction of alginate residue of the brown seaweed *Sargassum filipendula* for adsorbing cadmium metal ions [96]. After standard cleaning and powdering procedures, the raw biomass was activated via chemical impregnation using formaldehyde and hydrochloric solution in sequential order. The extraction process was carried out with the help of sodium carbonate solution. In short, laboratory tests showed a satisfactory outcome where up to 0.394 and 0.429 mmol/g of targeted adsorbate could be adsorbed at optimum temperatures of 293 and 303 K, respectively. In terms of the thermodynamic and kinetic examination, it was found that this adsorption run in a spontaneous and exothermic mode fitted to an external film model regarding mass transfer. In this case, the values of the kinetic constant were found to be 0.129, 0.064 and 0.066 min^−1^ at the initial concentrations of 1.0, 1.5 and 2.0 mmol/L, respectively, obeying Freundlich and the Dubinin-Radushkevich models.

### 6.6. Arsenic Metal Ions

Arsenic has long been identified as an extremely harmful toxicant. It can easily to pollute open waters due to either natural phenomena or man-made activities like mining and agricultural industries. While more complex technology can be implemented for its elimination from a watery environment, the use of bio-adsorbents could be a green solution for treating arsenic-contaminated water and wastewater. A study group became interested in examining a brown seaweed, namely *Sargassum muticum*, to solve the issue [97]. In order to enhance the ability of raw biomass to bind the targeted metal, the biomass was further washed and converted to iron-coated seaweed using FeCl_3_ solution. In depth analysis of the experimental data of the corresponding equilibrium and kinetic study regarding As (III) and As (V) suggested that maximum adsorptions of 4.2 mg/g and 7.3 mg/g could be obtained at pH 7 and 20 °C temperature, respectively. The uptake of As(III) tended to increase with the increase in pH. In contrast, the iron leaching went down when the pH increased due to the increasing solubility rate of iron hydroxide in the acidic environment. Moreover, As(III) adsorption underwent a redox reaction triggered by the involvement of As(III) oxidation to form As(V) along with the change of Fe(III) to Fe(II) when arsenate was the targeted adsorbate.

### 6.7. Mercury Metal Ions

Exposure to mercury even at trace amounts can have a huge detrimental effect on human health. This concern has encouraged some studies to further develop algae-based adsorbents for binding mercury ions. The removal of this metal using various microalgae species has given much information related to the potential of biomass. To start with, Fabre et al., selected six species of algae namely *Ulva intestinalis, Ulva lactuca, Fucus spiralis, Fucus vesiculosus, Gracilaria sp.,* and *Osmundea pinnatifida* as mercury adsorbents [98]. The washed algae biomasses were tested on a bench scale. It was found that the mercury adsorption mechanism followed the metabolically active adsorption process regarding metal incorporation onto the adsorbent surface. By obtaining an average metal removal efficiency of more than 80%, this study confirmed the competitive values of algae-based adsorbents. Bearing in mind that these adsorbents were used in their raw versions with no extensive modification, this indicates their beneficial characteristics as adsorbents.

Regarding the capability of being an alternative sorbent material, it is interesting to explore the diversity of algae and seaweed species. Therefore, a study simultaneously assessed three other species of algae having different natural colours, namely *Ulva lactuca* (green)*, Gracilaria gracilis* (red) and *Fucus vesiculosus* (brown), for their mercury uptake [104]. This study used these abundantly available biomasses in the marine environment for targeting mercury ions dissolved in solution as concentrations that were set within the range of 10–100 μg/L. A comprehensive analysis then disclosed that all selected biomasses could perform well, achieving adsorption capacities of more than 209 μg/g. Specifically, the comparative analysis of metal accumulation in living and dried biomass indicated that the green *Ulva lactuca* outperformed the red and brown algae. This conclusion referred to higher efficiency rate achieved at a faster contact time with more than 99% metal elimination using 500 mg/L of adsorbent dose. As a result, the treated water could meet the allowable standard of mercury content in drinking water.

### 6.8. Zinc Metal Ions

Despite its harmful effect, the heavy metal zinc also acts as a useful mineral at extremely low concentrations. Its solubility in water may lead to easy dispersion in wastewater streams derived from industrial sectors including mining and coal manufacturer. In relation to water pollution control, the presence of zinc can be managed well by adsorption technology, especially by using bio-adsorbents made of ubiquitous biomass like the marine seaweed *Ulva lactuca,* which has been proven effective for zinc removal from solution [99]. Senthilkumar and team used raw wastewater derived from phosphating industry as the aqueous medium for this purpose. Batch scale work via the adsorption technique was set up. With a maximum particle size by 1 mm, the cleaned dried biomass was contacted with the medium for the arranged time. Experimental analysis pointed out that nearly acid medium of pH 4.5 was the optimum condition allowing the adsorbent to bind more metal ions with a maximum adsorption capacity of 128 mg/g. In addition, HCl solution was found to perform remarkably well for regenerating the used adsorbent whereby around 99.6% of the adsorbed metal could be released from the surface and pores of the sorbent despite experiencing a 25% mass loss. This indicator is promising for further adsorbent usage in larger industrial wastewater treatment plants.

The appealing outcome of the previous work then increased the interest in conducting investigations on other algae for fabricating low-cost adsorbents. Jayakumar led a study to examine the adsorptive feasibility of the algae *Caulerpa scalpelliformis* that can be easily found in seawater environments [100]. After intensive washing, drying, and downsizing processes, a certain amount of nanometre-sized adsorbent was contacted thoroughly with a zinc polluted solution for about 60 min at a temperature of 30 °C. Primary parameters for the experimental work determined by implementing a Box-Behnken design gave pH 5.7, adsorbent dose of 1.5 g/L and a stirring speed of 150 rpm as the optimum variables. Therewith, by obeying Langmuir isotherm model, the metal uptake could reach 83.30 mg/g, while the kinetic analysis indicated that the result was better explained by a pseudo-second order model. From the perspective of the thermodynamic study, the adsorption process indicated a spontaneous and endothermic mechanism. The recyclable nature of the adsorbent was also reliable, as it had a relatively stable performance even after multiple adsorption cycles.

### 6.9. Multi-Metals Removal

Real industrial wastewaters mostly contain more than one heavy metal. The presence of multiple metals in raw wastewater is believed to strongly influence the accuracy of specific ion uptake by an adsorbent. Apart from that aspect, there are also various factors such as the level of nutrients, biomass growth, and the illumination rate that mostly affect metal adsorption processes in Nature. In order to understand the bio-adsorption process regarding multiple metals content, some investigations have been carried out using controlled working conditions. The potential of a brown algae, namely *Sargassum polycystum,* for the simultaneous adsorption of both cadmium and zinc ions was examined [101]. After massive washing to remove any impurities, the synthesized biomass particles were ground to an average size of 496.5 nm. Sample solutions were prepared by dissolving both Cd(NO_3_)_2_.4H_2_O and ZnSO4. Ultimately, a Box-Behnken design (BBD) was applied to predict the optimum conditions for metals removal and indicated that the optimum values of pH, adsorbent dose, and agitation speed for cadmium and zinc were 4.65, 1.8 g/L, and 76 rpm, and at 5.7, 1.2 g/L and 125 rpm, respectively. Furthermore, the metals uptake at equilibrium was inclined to better fit a Langmuir isotherm model meaning that both cadmium and zinc mostly adopted a single layer mechanism. By following a pseudo-second order model along with spontaneous and endothermic mode of thermodynamic inclination, the maximum adsorption capacities of cadmium and zinc were found to be 105.26 mg/g and 116.2 mg/g, respectively.

Another group reported the adsorption behavior for the elimination of multiple metal ions dissolved in water [102]. With the specification of alginate extraction residue in the preparation stage, the algal species named *Sargassum filipendula* was engaged for simultaneous removal of three prominent metal ions mostly detected in industrial wastewater like chromium, nickel, and zinc. After a standard preparation procedure, the adsorbent was further ground to obtain an average particle size diameter of 0.737 mm. The extraction was done by employing chemicals like formaldehyde, hydrochloric acid and sodium carbonate solutions in consecutive order to achieve more active sites on the adsorbent surface. This work used both simulated and raw effluents generated by tannery and leather manufacturers along with the entry and outlet sites of an urban water treatment plant. The initial characterization of collected raw industrial wastewaters denoted the existence of other metal ions such as magnesium, aluminum, iron, and lead at various concentration levels even though some metal elements were found to be lower than the standard detection limit. Batch experiments were carried out without pH adjustment. Overall, there was a higher affinity tendency for chromium metal in synthetic solution under the controlled conditions. In the supervised scenario, chromium achieved around 85% uptake, while zinc and nickel reached less than 40% removal with respect to the increase in temperature as a positive driver for adsorption at equilibrium. In contrast, the percentage of chromium elimination was significantly lower in the selected raw wastewaters. This can be linked to the higher competitiveness amongst the ions to occupy the binding sites on the adsorbent surface along with the presence of detected and undetected pollutants. Specifically, wastewater collected from an urban water station entry, for example, showed 80%, 70%, and 55% of removal efficiency for zinc, nickel, and chromium, respectively. A different outcome was noticed for wastewater derived from the outlet of the urban water treatment facility which just indicated around 42% and 5% of zinc and chromium removal, respectively, with no nickel removal detected. Furthermore, while tannery effluent only showed zinc and chromium removal by around 40% and 25%, respectively, leather wastewater showed a removal percentage of the three metals by 70%, 5%, and 22% for zinc, nickel, and chromium, respectively.

## 7. Regeneration and Disposal of Saturated Bio-Adsorbents

The literature shows that various attempts have been widely applied for regeneration of saturated or used adsorbents, including algal and seaweed-based adsorbents. Efforts were conducted in order to improve the lifespan of adsorbents by a recycling process via chemical activation after the first adsorption enabling the usage of cleaned adsorbent for further adsorption cycles. In this case, desorbing agents have been used to regenerate those saturated adsorbents. Some studies refer to alkalis as effective desorbing agents for desorbing heavy metals from chemically modified adsorbents, while acids are favorable for desorption processes of bio-adsorbent, especially, chelating the agent, ethylenediamine tetraacetic acid (EDTA). The ability of each biomass-based adsorbent to perform well in an adsorption process would reduce the time after several adsorption cycles until it reaches irreversible point. Once a saturated bio-adsorbent has worked unreliably for further usage due to an extreme decrease in its adsorption capacity, it then should be disposed of and managed properly based on the regulations established by relevant parties and labelled as hazardous waste [105].

## 8. Conclusions

It has been foreseen that the increasing activity of industry worldwide has led to a higher demand for green technologies for industrial water and wastewater treatment. This review covers the increasing interest in utilizing algae and seaweed-based adsorbents for heavy metals removal. As a low-cost biomass, algae and seaweed have promising reliability to be alternative adsorbent materials along with socioeconomical and environmental considerations. Other than that, some existing technologies for treating metal-contaminated water such as membrane filtration, coagulation and flocculation, biological and hybrid methods are also criticized to some extent. Furthermore, a comprehensive discussion of heavy metals’ characteristics, sources, and toxicity effects is properly reviewed supporting the importance of heavy metal elimination from water bodies. Based on the current literature, a clear conclusion can be drawn that this type of adsorbent has excellent potential for metal removal. Therefore, the recommendation of future study focusing on the treatment of raw industrial wastewaters on the large-scale could be encouraged by exploring other novel species of algae and seaweed, modifying biomass preparation methods prior to the adsorption process, and assessing novel ways of achieving low-cost adsorbent regeneration.

## Figures and Tables

**Table 1:** Allowable heavy metal levels in water.

Heavy Metals	Allowable Values (mg/L)		The Industrial Sources of Wastewater	Effect on Health
	WHO	EPA		
Mercury	0.01	0.05	Pharmaceutical, paper, pulp, ore, battery	Neurological diseases, paralysis, blindness
Lead	0.05	-	Battery, pipe, ceramic, glass production	Brain damage, anaemia, anorexia
Arsenic	0.01	0.05	Glass, mining, textile, paper, insecticides, phosphate fertilisers, mining, coal combustion	Lung and kidney cancer, liver tumour, nausea
Copper	1.0	0.25	Fertilizer, paints, pigments, tannery,	Liver and lung cancer, insomnia, osteoporosis, heart disease, headaches, seizures
Cadmium	0.003	0.005	Fertiliser, battery, power plants, mining, smelting, fuel combustion	lung cancer, kidney failure, bone lesions
Chromium	0.05	0.05 Cr(VI) 0.1 Cr(III)	Synthetic dyes, steel production, textile, ceramics	Lung cancer, haemorrhage, vomiting, severe diarrhoea
Nickel	0.015	0.2	Battery, mining, coinage, electroplating, glass, paints	Lung cancer, dermatitis, chronic asthma,
Zinc	3.0	1.0	Mining, steel fabrication, galvanisation, stabilisers, coal combustion	Gastrointestinal disorder, nausea, lethargy, neurological damage, loss of appetite

**Table 2:** The usage of algae/seaweed-based adsorbents for heavy metals removal.

Metal Ions	Adsorbent Material	Preparation/Activation Technique	Removal Efficiency (%)/Adsorption Capacity (mg/g)	Optimum Operating Conditions	Refs.
Lead	Seaweed *Turbinaria ornata*	Washing, drying (80 °C, 6 h), grinding (1–1.5 mm)	99.80%	Metal concentration 99.8 mg/L, agitation speed 250 rpm, and adsorbent dose 16.2 g/L	[88]
	Marine alga *Gelidium amansii*	Washing, drying (70 °C, 72 h), grinding (125 µm), mixing with distilled water, incubating and stirring (room temperature, 30 min), and final drying (70 °C, 72 h)	100%	Metal concentration 200 mg/L, temperature 45 °C, pH 4.5, adsorbent dose 1 g/L, contact time 60 min at static condition	[89]
Chromium	*Brown alga* *(Cystoseira barbata and Cystoseira crinite*)	Washing, drying (sunlight and oven (24 h, 60 °C), powdering (100–800 μm)	Cr(III): 70.70% (*C. barbata*), 73.34% (*C. crinite*) Cr (VI): 35% (*C. barbata*), 28% (*C. crinite)*	Cr(III): Metal concentration 100 ppm, contact time 120 min, pH 4.5, adsorbent dose 0.1 g/50 mL. Cr(VI): Metal concentration 100 ppm, contact time 24 h, adsorbent dose 100 mg/50 mL, pH 2.0).	[90]
	Brown algae *Sargassum dentifolium*	Washing, drying (50 °C, 24 h), grinding (0.3868 μm)	Cr(VI) 99.68%	Adsorbent dose 1.5 g/ 100 /mL, metal concentration 100 ppm, contact time flocculation state 1 h followed by 12 h static, pH 7.0, temperature 50 °C.	[91]
Nickel	seaweed *Sargassum filipendula*	Washing, drying (60 °C, 24 h), grinding (0.737 mm), extraction using formaldehyde and hydrochloric acid solutions, precipitation, redrying (60 °C, 24 h)	45%	Flow rate (dynamic system) 0.5 mL/min, metal concentration 1.0 mmol/L, Temperature 25 °C	[92]
	Algae biomass	Microalgae cultivation in batch mode with the help of Basal medium containing chemicals such as KNO_3_, Na_2_HPO_4_·12H_2_O, MgSO_4_·7H_2_O, FeSO_4_·7H_2_O, and CaCl_2_·2H_2_O, for creating a revolving algal biofilm. maintaining the culture in which half of the culture medium being exchanged with new medium each week for more than four years	95%	Metal concentration 100 mg/L, pH 7	[93]
Cobalt	Red alage *Gracilariacorticata*	Washing, drying, grinding, sieving (53 µm, 75 µm, 105 µm, 125 µm and 152 µm)	87.80%	Metal concentration 50 mg/L, pH 5, adsorbent dose 104 g/L, temperature 303 K or 29.85 °C	[94]
	Algae *Hypnea Valentiae*	Raw adsorbent: Washing, crushing, sieving (0.5–1mm), rewashing, drying (80 °C, 24 h). Modified adsorbent: All the above procedures followed by chemical impregnation (10% of formaldehyde, 1 h, room temperature, gentle mixing), filtering, rewashing, redrying (overnight, 60 °C)	Raw adsorbent 10.98 mg/g Modified adsorbent16.66 mg/g	Metal concentration 0.7 mg/L, temperature 30 °C, pH 6, adsorbent dose 2 g/L	[95]
Cadmi-um	Brown seaweed *Sargassum filipendula*	Washing, drying (60 °C, 24 h), milling, sieving (<1 mm). Alginate extraction using formaldehyde solution (0.4% *w/w*, 30 min), rinsing, mixing with HCl solution (0.1 moL/L, 2 h), followed by mixing with sodium carbonate solution (2% *w*/*v*, 5 h, 60 °C). Filtration to separate residue and the liquid phase with the solubilized alginate. Final washing with deionised water. Ethanol (1:1 *v*/*v*) for the alginate precipitation. Drying the residue and precipitated alginate (60 °C, 24 h), milling and sieving (0.737 mm)	0.43 mmol/g	Metal concentration 2 mmol/L, temperature 303 K, pH 3.5	[96]
Arsenic	Brown seaweed *Sargassum muticum*	Washing, drying (60 °C), grinding (5 and 12 mm) rewashing, redrying in overn (60 °C), mixing with 0.1 mol/L of FeCl_3_ 0.1 mol L^−1^ solution, stirring (200 rpm), precipitation using NaOH 10 mol/L for 24 h, filtration (pore size 2 mm), rewashing, redrying (60 °C)	≈100%	Metal concentration 2.5 mg/L, pH 7, temperature 293 K	[97]
Mercury	Macroalgae *Ulva intestinalis,* *Ulva lactuca*, *Fucus spiralis*, *Fucus vesiculosus*, *Gracilaria* sp., *Osmundea pinnatifida*.	Washing (tap water and synthetic seawater), maintaining macroalgae in aquarium with aerated seawater (a week, natural light approximately 12L:12D, room temperature 22 ± 2 °C), separation, freeze-drying	95% 90% 85% 80% 90% 80%	Metal concentration 1 mol/dm^3^, contact time 72 h, room temperature, pH 8.5	[98]
Zinc	Green seaweed *Ulva lactuca*	Washing, drying in an oven (24 h at 60 °C), crushing for smaller particle size (0.7–1 mm)	128 mg/g	Adsorbent dose 0.2 g/100 mL, contact time 6 h, agitation 150 rpm, temperature 31 ± 1 °C, pH 4.5	[99]
	Green macro algae *Caulerpa scalpelliformis*	Washing, drying under sun light (24 h), drying in an oven (50 °C, 48 h), particle downsizing measured by zetasizer (1326 nm)	83.3 mg/g	Adsorbent dose 1.5 g/L, contact time 1 h, agitation 150 rpm, temperature 30 °C, pH 5.7	[100]
Multi-metals	Brown algae *Sargassum polycystum*	Washing, drying under (24 h), multiple drying in an oven (24 h) (50 °C, 12 h), grinding, sieving (496.5 nm)	Cadmium: 86.20 Zinc: 92.90%,	Cadmium: pH: 4.65, adsorbent dose 1.8 g/L, agitation speed 76 rpm Zinc: pH 5.7, adsorbent dose 1.2 g/L, agitation speed 125 rpm	[101]
	Seaweed *Sargassum filipendula*	Washing, drying in an oven (60 °C, 24 h), sieving to obtain the medium particle diameter 0.737 mm. Extraction procedure: mixing biomass with formaldehyde solution (0.4 % *v*/*v*, 30 min), and washing, and mixing with hydrochloric acid solution (0.1 mol/L, 2 h), contacting to carbonate solution (2% *w*/*v*, 60 °C, 5 h). Alginate separation by vacuum filtration and ethanol for precipitation (1:1 *v*/*v*), further drying and sieving	Chromium0.864 mol/g Zinc 0.302 mmol/g Nickel 0.347 mmol/g	Temperature 50 °C, total metal concentration 1 mmol/L (17.33 mg/L of Cr; 19.56 mg/L of Ni; and 21.79 mg/L of Zn)	[102]
